# Event and Comment

**Published:** 1912-11-15

**Authors:** 


// DENTAL REGISTER.

Vol. LXVI. November 15, 1912. No. 11

### EVENT AND COMMENT.

OHIO STATE DENTAL SOCIETY. We print in another section the announcement of the Annual Meeting which this year will be held in Cincinnati instead of at Columbus as is customary. It has been many years since this society has met elsewhere than at Columbus, and this experiment will be watched with interest to see if a larger attendance or a more profitable meeting results. It will require a longer travel distance for some, and therefore those in distant parts of the State may not feel inclined to attend. Cincinnati however ought to supply a sufficient increase of attendance to more than make up for those who feel they can't go so far. The novelty of change will undoubtedly induce many to attend, and the special efforts of the Cincinnati men to provide an attractive program ought to make it well worth while traveling the extra distance to attend. In other states the meetings are generally held in different places each year, and the Illinois Society which has probably the largest attended meetings, meets each year in a different city. This method should induce many young men to become members who would not otherwise feel the need of joining the State Society, and it should also help to encourage the men to unite with their Local Societies which under the reorganization plan become component members of the State Society, and thus the way will be opened for their becoming members also of the National Society because of their membership in their local and State Organizations. If the Society could meet in the three cities, Cincinnati, Cleveland and Columbus in consecutive rotation, it would bring the State Society at least every three years near to all its members. We hope the meeting will justify the experiment.

NEW JERSEY HONORS MEEKER. A large number of the friends and admirers of Dr. Charles A. Meeker gave him a dinner, Oct 21st in Newark and presented him with a handsom diamond scarf pin with appropriate remarks as to the esteem in which they hold him because of his long devotion to the cause of dentistry in New Jersey and the Nation. The work of Dr. Meeker has not been confined to New Jersey, and the long list of admiring friends could have been considerably extended if members of the profession from other states had been consulted. A considerable local professional misunderstanding, of little cause, has disturbed the Jersey profession during the past year, but we believe it is now so happily adjusted that the regular order will prevail among the "hornets" unless some one pokes up the nest again. As usual Dr. Meeker got into the thick of the disturbance and although badly stung he seems to have come out triumphant in the esteem of a goodly number of his professional confreres judging by the long list of names who assisted in honouring him on this occasion. It is a fine thing for the brethren to dwell in unity or harmony, but many times it takes a real overturning of things to stimulate progress. The New Jersey men always were a lively bunch, and we are wondering what more they will do now that they are through with their house cleaning job. We shall expectantly wait for some movement that will attract the attention of the more conservative in other parts of the country.

